# Scarlatina Treated with Convalescent Serum

**Published:** 1914-06

**Authors:** 


**Scarlatina Treated with Convalescent Serum.** C. Rowe, Med. Klin., following the method of Reiss and Jungmann reported in 1912, claims good results.

